# Fontan-associated liver disease and hepatocellular carcinoma in adults

**DOI:** 10.1038/s41598-020-78840-y

**Published:** 2020-12-10

**Authors:** Tomomi Kogiso, Katsutoshi Tokushige

**Affiliations:** Department of Internal Medicine, Institute of Gastroenterology, Tokyo Women’s Medical University, 8-1 Kawada-cho, Shinjuku-ku, Tokyo, 162-8666 Japan

**Keywords:** Cancer, Gastroenterology

## Abstract

The Fontan operation creates a unique circulation, and is a palliative therapy for patients with single-ventricle congenital heart disease. Increased venous pressure and decreased cardiac output and hepatic venous drainage result in sinusoidal dilatation around the central veins. This causes congestion and hypoxia in the liver, leading to Fontan-associated liver disease (FALD). Non-invasive and invasive markers enable diagnosis and evaluation of the fibrosis status in chronic liver disease; however, these markers have not been validated in FALD. Additionally, regenerative nodules such as focal nodular hyperplasia (FNH) are frequently found. The severity of fibrosis correlates with the duration of the Fontan procedure and the central venous pressure. Cirrhosis is a risk factor for hepatocellular carcinoma (HCC), the annual risk of which is 1.5–5.0%. HCC is frequently difficult to diagnose and treat because of cardiac complications, coagulopathy, and congenital abnormalities. The mortality rate of FALD with liver cirrhosis and/or FALD-HCC was increased to ~ 29.4% (5/17 cases) in a nationwide survey. Although there is no consensus on the surveillance of patients with FALD, serial monitoring of the alpha fetoprotein level and imaging at 6-month intervals is required in patients with cirrhosis.

## Introduction

The Fontan procedure is a palliative operation for patients with single-ventricle congenital heart disease, in which the superior vena cava (SVC) drains into the distal right pulmonary artery (PA)^1^. The Fontan operation creates a unique circulation and has a broad spectrum of late outcomes. The procedure has been modified several times and improves the 30-year cardiac survival rate to > 80%^2,3^. However, Fontan-associated liver disease (FALD) and multiple liver nodules including benign tumors—such as focal nodular hyperplasia (FNH), hepatic adenoma, and hepatocellular carcinoma (HCC)—can develop postoperatively^4–6^. The diagnosis of HCC is often hampered by the characteristics of HCC arising from FALD (FALD-HCC), which can be similar to those of FNH^5^. In Japan, about 400 Fontan procedures are performed annually^7^, and the incidence of FALD and FALD-HCC is likely to rise with the frequency of the operation. Here, we review information on FALD and FALD-HCC and the future perspectives.

This study was conducted according to the principles of the Declaration of Helsinki and the ethical guidelines of the Tokyo Women’s Medical University Hospital (TWMU; Tokyo, Japan). The Institutional Review Board of the TWMU approved the study protocol. Informed consent was obtained from all participants.

### Fontan procedures

The procedure is typically indicated in children with tricuspid atresia, pulmonary atresia with intact ventricular septum, double-inlet left ventricle, hypoplastic left heart syndrome, double-outlet right ventricle, or complete atrioventricular septal defects^2,3^. There are three Fontan surgical techniques^2,8^. Classical Fontan was performed until around 1990, and the atriopulmonary connection was made by closing the atrial-septal defect and connecting the right atrium directly to the right PA (atriopulmonary method). This operation was later modified to a lateral tunnel procedure (intra-artrial lateral tunnel). The right atrium was baffled with an intraatrial patch and the SVC was directly connected to the right PA. After 2000, an extracardiac total cavopulmonary connection, which consists of a direct anastomosis of the SVC to the right PA and the insertion of an extracardiac conduit between the inferior vena cava (IVC) and the right PA, was constructed. The lateral tunnel method is associated with better short- and medium-term outcomes, compared to the extracardiac conduit method^9^.

The postoperative circulatory changes result from the following: (1) single ventricle circulation, (2) nonpulsatile pulmonary perfusion, (3) systemic venous hypertension, and (4) intracardiac scarring^10,11^. The term “Fontan failure” is generally applied to failure of the Fontan circulation causes the composite of all-cause mortality^12^. In other word, many complications were observed (Table 1a). The hemodynamic consequences of FALD vary based on the extent and stage of the liver involvement and may encompass the heart, lungs, and kidneys. Therefore, the hemodynamic status of failing Fontan should be evaluated when considering treatment for FALD (Table 1b)^13,14^.

**Table 1:** Phenotypes of patients with a failing Fontan.

Condition	Incidence	Manifestations		
**(a) Early and late complications of patients with a failing Fontan**^12^				
Early failure	3%	Low cardiac output, pleural effusions, chylothoraces, ascites, hepatomegaly		
**Late failure** Lymphatic dysfunction, PLE	2–13%	Ascites, peripheral edema, pleural effusions, diarrhea, malabsorption of fat, hypoalbuminaemia		
Plastic bronchitis	< 2%	Tachypnoea, cough, wheezing, expectoration of bronchial casts		
Primary ventricular dysfunction	− 7 to 10%	Progressive exercise intolerance, AV valve insufficiency, hepatomegaly, ascites		
Progressive increase in pulmonary resistance	Unknown	Hypoxaemia		
Hepato-renal insufficiency	Unknown	Renal dysfunction		
Hepatic complication	41% (57/139)^19^ Liver cirrhosis and/or HCC: total 1.15%^49^ HCC: 1.5–5.0% annually in cirrhosis^49^	Hepatomegaly, ascites, splenomegaly, HCC		

Description	Type I (systolic heart failure)	Type II (diastolic heart failure)	Type III (non-cardiac failure) Relevant to discussion of FALD	Type IV (plastic bronchitis and PLE) Fontan failure with lymphatic abnormalities
**(b) Hemodyamic status of patients with a faling Fontan**^13,14^				
Systolic function	**↓**	**→**	**→**	**→**
Ventricular EDP	**↑**	**↑**	**→**	**↓** or →
Cardiac output	**↓** or →	**↓** or →	**→**	**→**
Systemic vascular resistance	**↑**	**↑**	**↓** or →	**↓** or →

*AV* atrioventricular, *EDP* end-diastolic pressure, *HCC* hepatocellular carcinoma, *FALD* Fontan-associated liver disease, *PLE* protein-losing enteropathy.

### Fontan physiology

**Figure 1:**
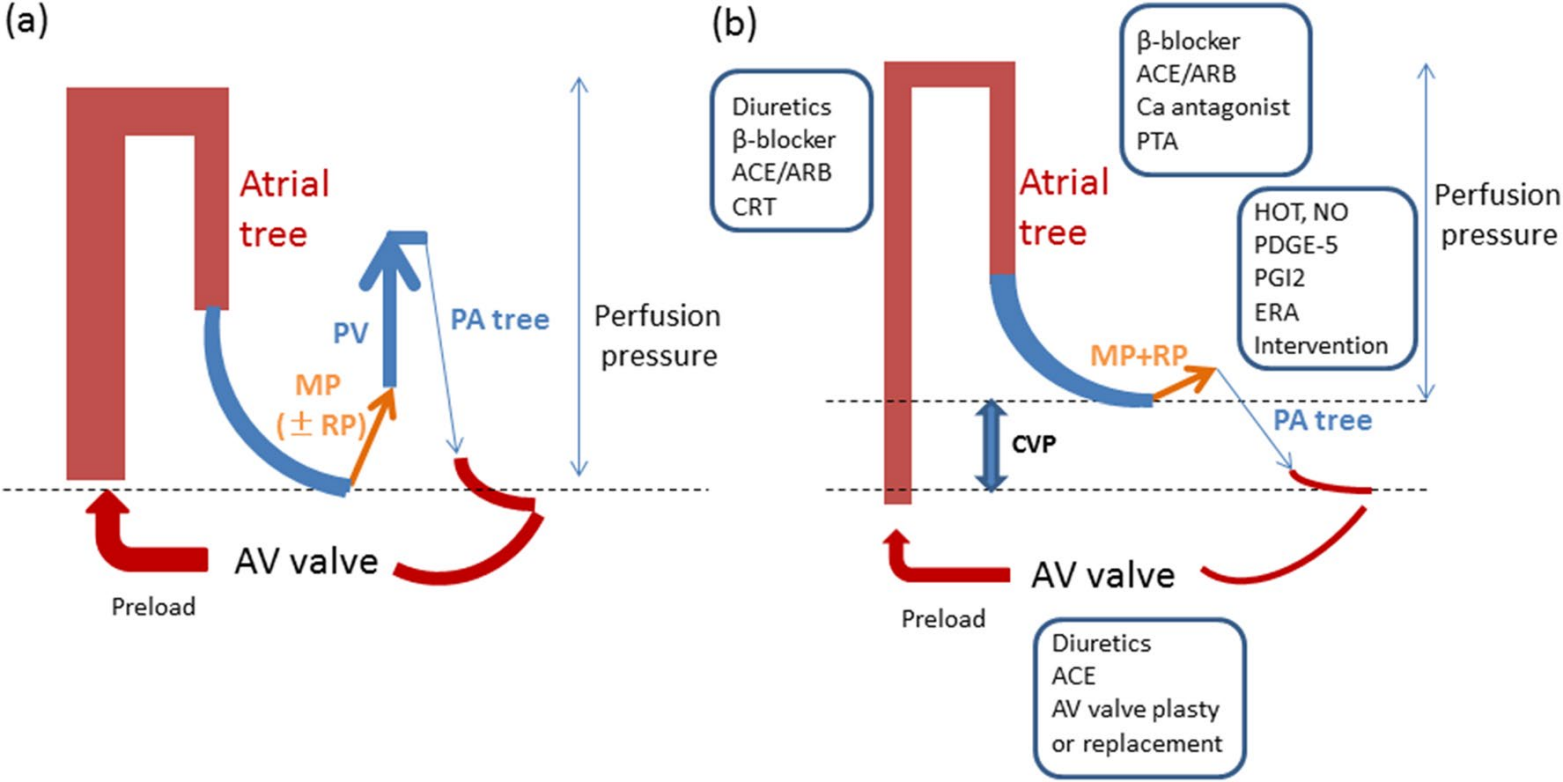
Hemodynamic changes after the Fontan procedure and treatment thereof. Central venous pressure frequently increases after Fontan surgery (**b**) compared to normal (**a**). ACE, angiotensin-converting enzyme; ARB, angiotensin receptor blocker; AV, atrioventricular; Ca, calcium; CRT, cardiac resynchronization therapy; CVP, central venous pressure; ERA, endothelin receptor blocker; HOT, home oxygen therapy; MP, muscle pump; NO, nitric oxide; PA, pulmonary artery; PV, pulmonary ventricle; PDGE-5, phosphodiesterase 5 inhibitor; PGI2, prostaglandin I2; PTA, percutaneous transluminal angioplasty; RP, PA resistance.

**Figure 2:**
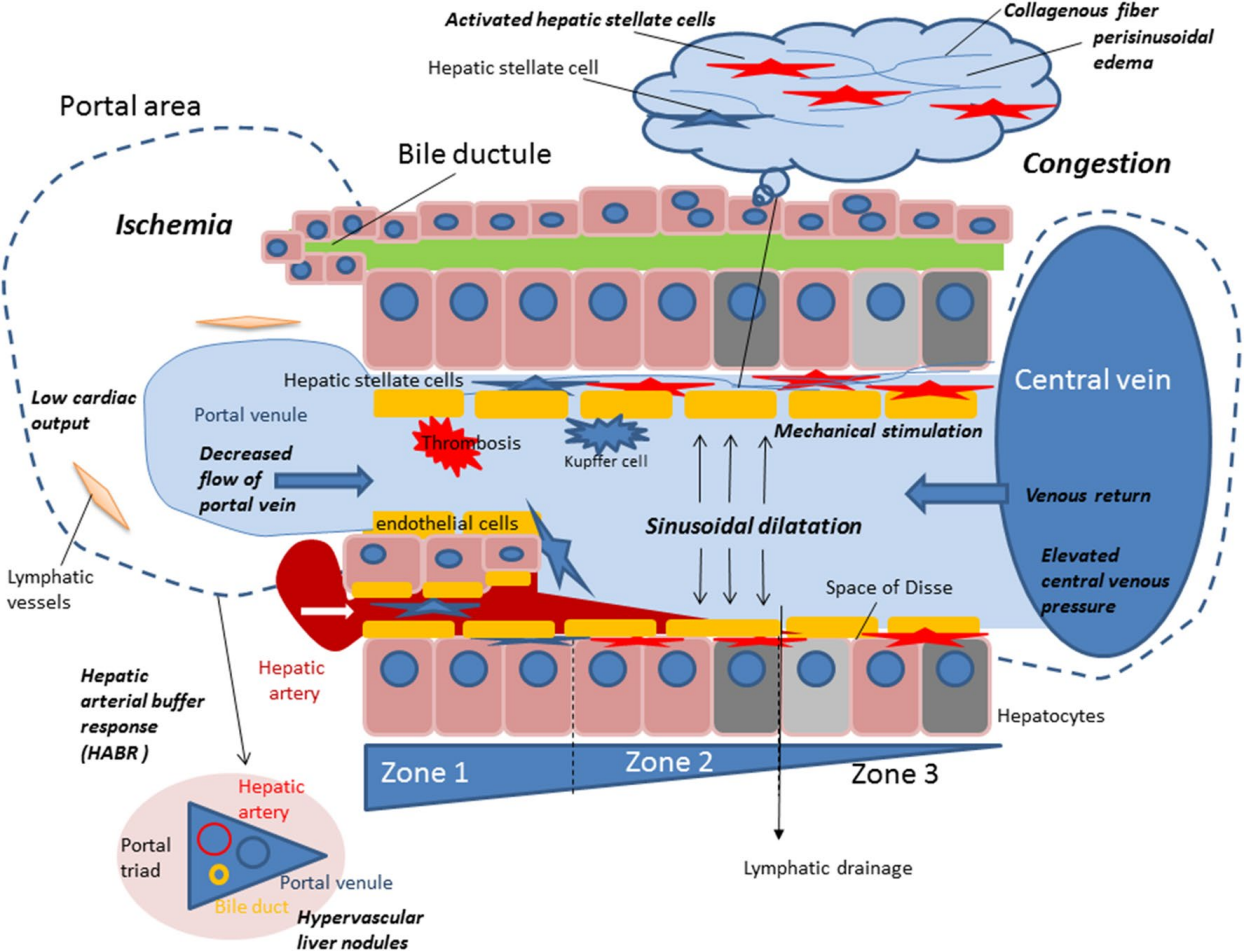
Fontan physiology. Increased venous pressures and decreased cardiac output and hepatic venous drainage result in sinusoidal dilatation around the central veins. This causes congestion and hypoxia in the liver, leading to Fontan-associated liver disease (FALD). Hepatic stellate cells are activated and collagenous fibers are produced. Decreased portal flow induces a hepatic arterial buffer response (HABR) and hypervascular nodules are formed mainly in peripheral areas of the liver.

Central venous pressure (CVP) typically increases after Fontan surgery (Fig. 1b), as compared to normal (Fig. 1a). The hepatic venous pressure and pressure load on the central vein in the hepatic lobule cause congestion of the liver^15,16^. Mutual buffering between the portal blood flow and the hepatic artery blood flow is known as the hepatic arterial buffer response (HABR)^15^ (Fig. 2). When the portal blood flow decreases, the hepatic artery is dilated to regulate the hepatic blood flow. Thus, the oxygen supply to the liver tissue is constantly maintained. Mechanical stimulation caused by dilation of the hepatic sinusoids, thrombus formation as a result of abnormal coagulation, and congestion and hepatocyte hypoxia are observed^17^. HABR increases the hepatic artery blood flow and hypernodular lesions may form in hypoxic areas, particularly in peripheral areas of the liver. Additionally, hypoxia and thrombosis within the sinusoids promote the activation of hepatic stellate cells (HSCs) and the production of fibronectin, leading to portal and sinusoidal fibrosis. Perisinusoidal edema and ischemic liver cause fibrosis progression without centrilobular inflammation. When fibrosis further progresses, cross-linking fibrosis mainly connecting the central zones is observed histologically, and a fibrous septum is formed, a finding of cirrhosis. Congestion causes the formation of an inverted image of the hepatic lobules (‘reverse lobulation’; an image in which the portal area is located in the center and the hepatocyte population is surrounded by a congestion zone). Postsinusoidal hepatic outflow obstruction lead to accumulation of ascites presenting a high protein level. The ascites showed a protein level of > 2.5 g/dL and the serum ascites an albumin gradient of > 1.1 g/dL^18^. Ascites may be caused by increased sinusoidal pressure and/or impaired lymphatic drainage^4^.

### Prevalence and diagnosis of FALD

Baek et al. evaluated 139 patients who underwent Fontan surgery and found hepatic complications in 57 (41%)^19^. In the blood test, transaminase levels were typically within the normal range or mildly elevated in FALD (Table 2). The γ-glutamyltransferase (GGT) level was mildly elevated (median = 69 U/L) in 75% of patients^20^, but this was not correlated with histological severity. Camposilvan and colleagues reported the following complications in 34 patients (average age = 14.7 years): hepatomegaly in 53%, splenomegaly in 9%, transaminase abnormality in 30%, GGT elevation in 61%, elevated serum bilirubin in 32%, abnormal coagulation in 58%, and protein losing enteropathy (PLE) in 19%^21^.

**Table 2:** Non-invasive and invasive assessment of FALD.

Variable	Findings	Value of estimating liver cirrhosis	Availability and warning
**Biomarkers**			
AST, ALT, GGT, T-BIL, platelet count	Elevated	Varies	
	Decreased	Not indicated the cutoff value	
Type IV collagen, hyaluronic acid, and P-III-P	Elevated Elevated Elevated	Not indicated the cutoff value > 46 ng/mL^20^ Not indicated the cutoff value	PPV 33.3%, NPV 38.5%
M2BPGi	Normal	**–**	
FibroSURE	Elevated α2-macroglobulin, haptoglobin, apo-lipoprotein A1, bilirubin, GGT, and alanine transaminase along with age and gender to measure fibrosis activity of the liver	> 0.74^20^	PPV 33.3%, NPV 52.6% No correlation with histological findings
AST/ALT ratio	AST, ALT	< 1 normal^19^	
AST-to-platelet ratio index (APRI) score	AST, ALT, platelet count	> 2^39^, > 1.5^44^	
The MELD XI score	Bilirubin, creatinine	> 12.0^44^	Correlated with histological findings. Non cut-off points with adequate sensitivity and specificity
Fibrosis-4 (FIB-4) index	AST, ALT, platelet count, and age	> 3.25^39^, > 1.45^44^	
The Forn index	GGT, platelet count, age, and cholesterol	**> **6.9^45^, > 4.2^44^	
The VAST score	Varices, ascites, splenomegaly, thrombocytopenia	**≥ **2^47^ portal hypertension	
Ultrasound^13,31,79^	Normal or slightly hypoechoic at the early stage Heterogeneous hyperechoic parenchymal pattern and surface nodularity Caudate lobe hypertrophy Irregular parenchymal fatty infiltration with perivascular distribution	Nodular liver surface Increased echogenicity Irregular borders Splenomegaly Ascites Collateral circulation	Detection of cirrhosis; sensitivity 88%, specificity 82–95%^80^
CT^13,31,79^	Abnormal parenchymal enhancement Reticular pattern (i.e., peripheral diffuse patchy enhancement) in the delayed phase Zonal enhancement (i.e., altered enhancement of the liver periphery) is correlated with lower hepatic vein pressures and a lower likelihood of cardiac cirrhosis Surface nodularity Caudate lobe hypertrophy	Reticular pattern Irregular or nodular liver surfaces Splenomegaly Ascites Collateral circulation	Detection of cirrhosis; sensitivity 77–84%, specificity 53–68%^80^
MRI^13,31,79^	Increased T2-weighted and diffusion-weighted signal with reduced T1-weighted signal intensity in the periphery of the liver Reticular or mosaic patterns of diminished enhancement (i.e. "frog spawn" appearance) on Gd-EOB-MRI	Splenomegaly Ascites Collateral circulation	Detection of cirrhosis; sensitivity 87%, specificity 92%^80^
**Elastography** Transient elastography (TE, fibroscan) SWE ARFI MRE		> 17.6 kPa^81^ > 19.8 kPa^82^ > 1.55 m/s^33^ > 4.9^83^	Hepatic congestion alone can increase stiffness Direct comparison of types of elastography is lacking in FALD
HPVG	The gradient between the hepatic and portal veins	> 5 mmHg; portal hypertension^13^	
Pathological findings	Gross appearance of ‘nutmeg liver’^18^ Liver sinus fibrosis 94%, centrilobular necrosis 33%, pericentral fibrosis 79%, and portal vein fibrosis 76%^35^ Fibrous septa bridging central vein ‘reverse lobulation’^18^	fibrotic septa separated regenerative nodules	Detection of cirrhosis by liver biopsy; sensitivity 80–100%, specificity 80–100%^80^

*ALT* alanine aminotransferase, *ARFI* acoustic radiation force impulse, *AST* aspartate aminotransferase, *CT* computed tomography, *FALD* Fontan-associated liver disease, *Gd-EOB-MRI* gadolinium ethoxybenzyl diethylenetriamine pentaacetic acid -enhanced magnetic resonance imaging, *GGT* γ-glutamyl transferase, *HPVG* hepatic venous pressure gradient, *M2BPGi* mac-2 binding protein glycosylation isomer, *MELD* model for end-stage liver disease, *MRE* magnetic resonance elastography, *NPV* negative predictive value, *P-III-P* procollagen-III-peptide, *PPV* positive predictive value, *SWE* shear wave elastography, *T-BIL* total birirubin, *VAST score* varices, ascites, splenomegaly, thrombocytopenia.

Fibrosis markers, such as hyaluronic acid and type IV collagen 7S, are typically increased in the presence of liver complications and are useful for evaluating FALD^20,22^. Procollagen-III-peptide (P-III-P) is more susceptible to inflammation and it is said diagnosing the stage of fibrosis is inferior than hyaluronic acid or type IV collagen^22^. Shimizu et al. suggested hyaluronic acid and GGT as markers of the progression of liver fibrosis in Fontan patients^23^. While mac-2 binding protein glycan isomer (M2BPGi) is a useful marker of chronic hepatitis, particularly in patients with hepatitis C virus (HCV) infection^24^. However, it is unlikely to rise of M2BPGi in the cases of Fontan, because FALD is not accompanied by inflammation.

In the imaging, the liver may appear normal on radiological examination or slightly hypoechoic on ultrasound at the early stage of congestive hepatopathy. As fibrosis develops, a coarse heterogeneous hyperechoic parenchymal pattern and surface nodularity become evident^18^. The liver is often enlarged with caudate lobe hypertrophy, similar to Budd-Chiari syndrome. Irregular parenchymal fatty infiltration of a perivascular distribution can be seen on ultrasound. Contrast-enhanced ultrasound (CEUS) shows heterogeneous and decreased enhancement of the liver in the portal venous phase^25^. Hepatic vein waveforms assessed by doppler ultrasound change in accordance with liver fibrosis progression^26^.

In computed tomography (CT), hepatic fibrosis may be seen as reticular pattern on delayed-phase CT (i.e., peripheral diffuse patchy enhancement)^13^. Zonal enhancement (i.e., altered enhancement of the liver periphery) is correlated with lower hepatic vein pressure and a lower likelihood of cardiac cirrhosis^13^. On magnetic resonance imaging (MRI), there are areas of increased T2-weighted and diffusion-weighted signals with reduced T1-weighted signal intensity in the periphery of the liver, corresponding to areas of abnormal contrast enhancement^27^. Gadolinium ethoxybenzyl diethylenetriamine pentaacetic acid-enhanced MRI (Gd-EOB-MRI) revealed a characteristic reticular or mosaic pattern of diminished enhancement (i.e., ‘frog spawn’ appearance)^28^. The apparent diffusion coefficient (ADC) calculated by diffusion-weighted imaging (DWI) enables estimation of the degree of hepatic fibrosis^25^.

Assessment of liver stiffness by transient elastography (TE), acoustic radiation force impulse (ARFI) elastography, and magnetic resonance elastography (MRE) enables evaluation of hepatic fibrosis^29–31^. However, it was not correlated with the histopathologic findings at a single time point^32^. ARFI and TE might be useful for monitoring liver stiffness in patients with Fontan physiology. The mean shear wave propagation velocity in liver tissue by ARFI elastography in Fontan patients was 1.86 ± 0.5 m/s in 21 patients^33^. Of that, 76% of patients had a value over the cirrhosis threshold of 1.55 m/s. In contrast, the mean shear wave propagation velocity was significantly lower in patients who had undergone heart transplant^31^. However, TE/shear-wave elastography (SWE) cannot distinguish hepatic congestion from fibrosis^34^. While MRE reportedly enables evaluation of liver fibrosis^30^, its utility in FALD needs to be evaluated.

The gross appearance of the liver is termed ‘nutmeg liver’ in patients with FALD^18^. The pathological findings showed liver sinus fibrosis in 76%, centrilobular necrosis in 33%, pericentral fibrosis in 79%, and portal vein fibrosis in 52% of cases^35^. FALD showed dilatation and fibrosis of hepatic sinusoids, fibrosis of the portal area, and no inflammation^36,37^. Pathological evaluation of the liver via percutaneous or transvenous biopsy is the gold standard for assessing the degree of fibrosis; however, obtaining liver samples is difficult and there is a risk of bleeding. Also, the role and timing of the initial and follow-up liver biopsies in this population are unclear.

### Diagnosis of liver cirrhosis by invasive and non-invasive biomarkers in FALD

Cardiac cirrhosis results from prolonged passive liver venous congestion secondary to right-sided congestive heart failure and is defined as stage-4 fibrosis on liver biopsy^38^. At this stage, obtaining liver samples is hampered by the risk of bleeding and ascites accumulation. As another invasive examinatioin, transjugular measurement of the hepatic venous pressure gradient (HVPG, the gradient between the hepatic and portal veins) of > 5 mmHg is suggestive of sinusoidal portal hypertension^13^. The HVPG is useful for differential diagnosis of ascites of cardiac origin (normal HVPG) or hepatic origin (elevated HPVG)^39^.

Instead of these invasive examinations, typical imaging findings of the liver, formation of esophageal and gastric varices, ascites accumulation, and splenomegaly can facilitate diagnosis of liver cirrhosis. Moreover, several scores are used to assess patients with end-stage liver disease^40^. The model for end stage liver disease (MELD) XI score, which is based on the serum bilirubin and serum creatinine levels, may be predictive of the outcomes of Fontan patients^41^. A recent retrospective review revealed a positive correlation between the MELD-XI score and hepatic fibrosis scores on pathology (correlation coefficient = 0.4; *p* = 0.003)^42^. Although, a receiver operator characteristic analysis did not identify a score cutoff with adequate sensitivity and specificity^42^, patients with a MELD-XI score of ≥ 19 had a higher mortality rate^43^.

Proprietary tests such as FibroSURE, which includes assessment of multiple serum markers, have been validated only in patients with HCV and rely on inflammatory markers that are unlikely to be relevant in FALD. FibroSure for identifying evolving or established cirrhosis when compared to liver biopsy had a positive predictive value (PPV) of 33.3% and a negative predictive value (NPV) of 52.6%^20^. A hyaluronic acid level of > 46 ng/mL is indicative of liver cirrhosis and the PPV and NPV were 33.3% and 38.5%, respectively^20^.

Besides, the aspartate aminotransferase (AST)/alanine aminotransferase (ALT) ratio, AST-to-platelet ratio index (APRI) score, Forns index, and fibrosis-4 (FIB-4) score are reported as markers of fibrosis in FALD^19,39,44,45^. While the FIB-4 index includes age and has lower predictive utility among young adults with other liver disease^46^. In contrast, the VAST score is used to evaluate portal hypertension according to the liver-related complications^47^. A VAST score of ≥ 2 was reported to be significantly related to major adverse events (odds ratio = 9.8, 95% confidence interval [CI] = 2.9–32.7)^47^.

### Treatment for FALD

Medical therapies specific for FALD are not available. Nevertheless, preventive, medical, surgical, and transplant strategies beneficial for similar disease processes may be applicable in FALD^14,16,48^. Prior to initiating a treatment strategy, it is important to improve cardiac output and/or raise the Fontan pressure (Fig. 1b).

Regarding treatments for liver cirrhosis, ursodeoxycholic acid (UDCA), lactulose, kanamycin, and heart–lung transplantation were used in a nationwide study^49^. Generally, UDCA treatment decreased the elevation of liver enzymes; however, the long-term benefit for FALD was uncertain. A branched-chain amino acid (BCAA), an antibacterial agent (rifaximin), carnitine, and/or synthetic disaccharide may be used for FALD to prevent encephalopathy by reducing the ammonia level. For ascites, since the renin-angiotensin system is upregulated in liver cirrhosis, spironolactone is used prior to frosemide as diuretics^50^. Additionally, tolvaptan, a highly selective vasopressin-V2 receptor antagonist, is used to treat cardiac failur and refractory ascites^50^. In more severe cases of ascites, cell-free and concentrated ascites reinfusion therapy (CART) or ascites drainage therapy can be considered. Infusion volume load and fever should be care. Control of ascites is frequently hampered by postsinusoidal outflow obstruction.

In patients with more severe disease, cardiac transplantation was selected for cases with no evidence of liver cirrhosis. Overall, the 5- and 10-year survival rates were 72% and 69%, respectively, after cardiac transplantation^51^. Combined heart-liver transplantation should be considered in severe cases^52^. It reportedly has a favorable outcome, with a 10-year survival rate of 83%^52^.

### Prevalence, characteristics, and diagnosis of HCC

The prevalence of liver nodules is reportedly 29.6% (95% CI 23–37%) on ultrasound and 47.7% (95% CI 39–56%) on CT/MRI^53^. Nodules were usually hyperechoic (76.5%), round-shaped (> 80%), hyperenhancing in the arterial phase (92%) and located in the liver periphery (75%) (Table 3)^53^. In a study based on nationwide surveys of FALD-HCC, 31 HCC cases (1.15%) were detected among 2,700 cases who had undergone the Fontan procedure^49^. In multicenter case studies, 33 HCC cases (1.3%) were observed among 2,470 patients who had undergone the Fontan operation^54^. The estimated annual incidence is 1.5–5.0% in patients with liver cirrhosis^11,53,55^. Case reports of FALD-HCC are listed in Table 4. In our cohort, HCC was diagnosed in 12 cases (9.8%) at a median age of 32.5 years (range: 20.6–46.1 years), and the median interval between the Fontan procedure and diagnosis was 21.3 years (range: 3.7–31.2 years), an incidence of 2.9%^56^.

**Table 3:** Characteristics of liver nodules in FALD^53^.

Modality	Ultrasound	CT/MRI
Hepatic nodules	3/49 (6.1%)^31^ 45/152 (29.6%)	CT; 14/44 (31.8%), MRI; 19/48 (39.6%)^31^ CT (n = 37)/MRI (n = 93); 62/130 (47.7%)
	Ultrasound (n = 152)	CT (n = 37)/MRI (n = 93)
Medium size of nodules	11 (6–18) mm	9 (6–12) mm
**Number of nodules**		
(1/2/3/more)	15 (33.3%)/11 (24.4%)/10 (22.2%)/4 (8.8%)	30 (23.1%)/15 (11.5%)/6 (4.6%)/11 (8.4%)
Nodular parenchyma with countless micronodules	5 (11.1%)	
**Shape**		
Round	85 (83.3%)	140 (90.3%)
Ellipsoidal	7 (6.8%)	6 (3.9%)
Irregular	5 (4.9%)	9 (5.8%)
Periphery location	68 (66.6%)	116 (74.8%)
Other characteristics	<Echogenicity> Hyperechoic 78 (76.5%) Isoechoic 15 (14.7%)	<CT> liver imaging reporting and data system 1:10 (6.4%), 2:24 (15.5%), 3:92 (59.4%), 4:6 (3.9%), 5:5 (3.2%), Unclassified: 18 (11.6%) <MRI> T1-weighted MRI isointense 76 (71.7%) T2-weighted MRI isointense 85 (80.2%)
Arterial-phase enhancement/wash out		143 (92.3%)/11 (7.1%)

*CT* computed tomography, *MRI* magnetic resonance imaging.

**Table 4:** Characteristics of patients with Fontan-associated liver disease-hepatocellular carcinoma (FALD-HCC).

Case	Gender	Age at HCC detection (years)	Post Fontan (years)	Complications	AFP (ng/mL)	Treatment	Pathological diagnosis	Prognosis/cause of death
Ghaferi and Hutchins^5^	Male	24	18	ASD, VSD, cirrhosis	ND	–	+	Died, ruptured HCC
Ewe^84^	Male	29	19	ASD, VSD, cirrhosis	4674	Oral chemotherapy	–	Alive
Saliba et al.^85^	Female	27	23		162.7	Chemotherapy	–	Died after 1 year
	Female	28	18		788.9	Sorafenib	–	Died after 1 year
Rosenbaum et al.^86^	Female	13	2		3340 (ug/L)	TACE	–	Alive
Asrani et al.^6^	Female	32	–	Cirrhosis	700	TACE	–	Waiting for CHLT
	Male	24	–	PVTT, ascites, gastric varices	5000	–	Well-differentiated	Died, metastasis
	Male	33	–		630	Radioembolization	–	Died, hepatic artery pseudoaneurysm ruptured
	Female	42	–	HCV, advanced fibrosis	106	TACE	–	Waiting for CHLT
Elder et al.^87^	Male	51	28	Atrial arrhythmias, ascites, pleural effusions	Normal	Local ablation	–	Heart transplantation Cancer free
Wallihan et al.^88^	Male	15	11		–		Fibrolamellar HCC	–
Rajoriya et al.^89^	Female	41	22	Situs invers	–	Sorafenib	–	Died
Weyker et al.^90^	Female	23	22		–	Liver resection	+	Alive
Yamada et al.^91^	Male	15	14		2	TACE	–	Died after 2 years
Kwon et al.^92^	Male	32	23	Tachycardia	13 (μg/L)	Liver resection	Fibrolamellar HCC	Cancer free
Oh et al.^93^	Female	16	14	Sinus bradycardia	211,580	Chemotherapy	–	Lung metastasis Died after 2 months due to hematemesis
Takuma et al.^94^	Female	29	19	Situs inversus	117.1	Liver resection	Poorly differentiated	Alive, cancer free
Josephus Jitta et al.^80^	Male	30	18	ASD, VSD	20,740 (μg/L)	BSC	–	Died Metastasis to Lungs
	Female	42	32	ASD, VSD, early cirrhosis	2996 (μg/L)	Liver resection + RFA	+	Alive
	Female	48	34	ASD, VSD, cirrhosis	865 (μg/L)	BSC	–	Died
Lo et al.^95^	Female	24	23	Atrial tachycardia, cirrhosis	50,000 (ng/dL)	Liver resection TACE	Moderately differentiated Lymphovascular invasion	Died after 6 months, HCC recurrence
Mazzarelli et al.^66^	Female	28	18		8	Sorafenib	Moderately differentiated, HCC with vascular infiltration	Alive
	Female	20	18		12,000	TACE	–	Alive
	Male	21	17	VSD	4	TACE	–	Waiting for CHLT
Angelico et al*.*^64^	Female	33	6		3005	Laparoscopic liver resection		Sorafenib treatment combined with TACE. After downsizing, CHLT was performed
Ogasawara et al.^67^/Sagawa et al.^56^	Female	27	21	Polysplenia, heterotaxy	1622	PBT	–	No recurrence
Sagawa et al.^56^	Male	31	21	Cerebral infarction	4	BSC	–	Died due to heart failure
	Female	36	30	HCV	5520	BSC	–	Died due to liver and heart failure
	Male	46	21	HCV, post MVR	743	BSC	Well differentiated	Died due to heart failure
	Female	23	19	PVTT	14,867	Hepatic arterial infusion chemotherapy	–	Died due to liver failure and HCC
	Male	22	4	SSS	7	TACE	–	No recurrence
	Male	34	30		7	Liver resection	Poorly differentiated	Unknown
	Male	42	29	SSS	7	PBT	–	Died due to bleeding of metastatic HCC in the chest cavity
	Female	33	27	SSS	8786	PBT	–	Lung metastasis
	Male	20	15	PLE, polysplenia	5	PBT	Well differentiated	Intrahepatic recurrence
	Male	27 30	22 25		78 3901	TACE PBT, liver resection	Well differentiated Poorly differentiated	Intrahepatic recurrence PBT and liver resection were undertaken for recurrence
Nemoto et al.^63^/Sagawa et al.^56^	Female	36	31	Polysplenia	81,663	Liver resection	Poorly differentiated	Lung metastasis
Yokota et al*.*^65^	Male	18	6		3	Laparoscopic liver resection	Well-differentiated	Alive

*AFP* alpha fetoprotein, *ASD* atrial septal defect, BSC, best supportive care, *CHLT* combined heart-liver transplant, *HCC* hepatocellular carcinoma, *HCV* hepatitis C virus, *MVR* mitral valve replacement, *ND* not detected, *PBT* proton beam therapy, *PLE* protein-losing enteropathy, *PVTT* portal vein tumor thrombosis, *RFA* radiofrequency ablation, *SSS* sick sinus syndrome, *TACE* transcatheter arterial chemoembolization, *TAE* transcatheter arterial embolization, *VSD* ventricular septal defect.

Liver nodules are missed on ultrasound in 30% of cases^31^. Contrast-enhanced CT and Gd-EOB-MRI enable detection of FALD-related HCC, however, 14% of FNH cases show portal/delayed washout, which is also present in patients with HCC^57^. The sensitivity of positron emission tomography (PET)-CT scan is only 55% for diagnosis of HCC^58^. Wells et al. demonstrated that mosaic architecture and an elevated alpha fetoprotein (AFP) level are associated with HCC, especially an AFP level of ≥ 400 ng/mL^59^. Some HCC cases are difficult to diagnose because of the lack of an increased AFP level. Again, a large proportion of the patients were treated with warfarin potassium and obtaining a tumor biopsy sample for diagnosis was problematic. Also, it affected the level of des-gamma-carboxy prothrombin (DCP), a marker of HCC, in turn hampering the diagnosis of HCC. Therefore, a new marker for FALD-HCC is needed.

**Figure 3:**
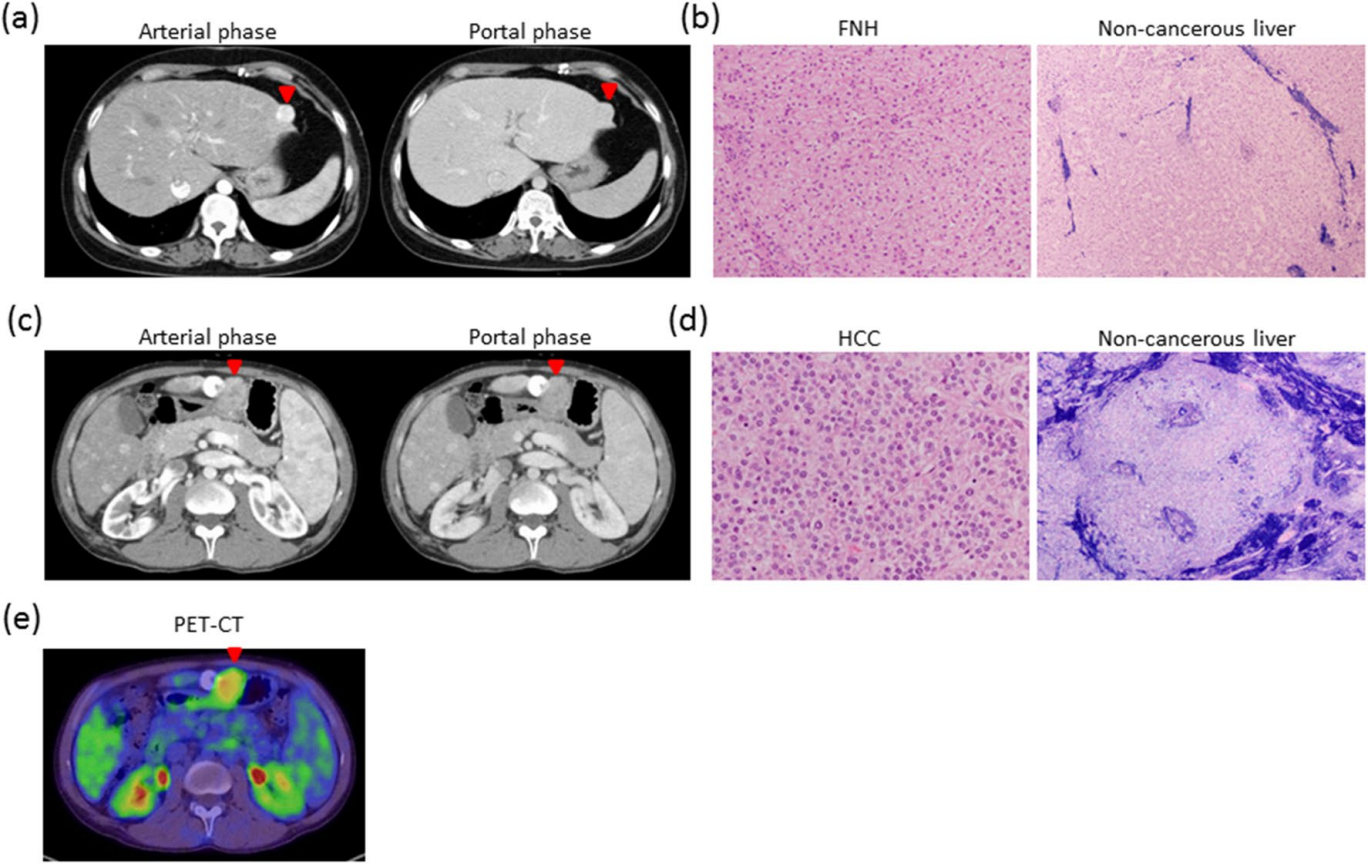
Liver tumors arising from FALD. A 37-year-old female had a complicated hypervascular tumor periphery on enhanced abdominal CT (**a**). HCC could not be ruled out. Ultrasound did not detect the nodule and MRI could not be performed because of a pacemaker. Surgically resected specimen revealed FNH (**b**, left H&E staining). Non-cancerous liver specimen showed sinusoidal dilatation and mild fibrosis (b, right; Victoria blue H&E staining). A 30 year-old male had a hypervascular tumor on enhanced abdominal CT scan (**c**). The tumor was more enhanced at the late arterial phase. HCC was treated with TACE and PBT; however, it was not completely cured. Finally, surgery was selected and HCC of confluent multinodular type and poorly differentiated was diagnosed (**d**, left; H&E staining). The tumor was positive by PET-CT (**e**). A non-cancerous liver specimen showed cirrhosis (**d**, right; Victoria blue H&E staining). CT, computed tomography; FALD, Fontan-associated liver disease; FNH, focal nodular hyperplasia; H&E, hematoxylin and eosin: HCC, hepatocellular carcinoma; MRI, magnetic resonance imaging; PBT, proton beam therapy; PET, positron emission tomography; TACE, transcatheter arterial chemoembolization: Tc-99m GSA, technetium-99m diethylenetriamine pentaacetic acid galactosyl human serum albumin.

There are no reports of the misdiagnosed rate of patients with FALD-HCC. Almost all investigations have been case reports, and large studies are limited. In our 124 cases, we detected 77 (62.1%) cases with hyperechoic lesions on ultrasound. Twelve patients were diagnosed with HCC^56^. An increase in AFP was observed in seven cases. Five cases were finally diagnosed with HCC by imaging and the clinical course. We experienced several HCC cases that were difficult to distinguish from FNH. Case 1: A 37-year-old female had a complicated hypervascular tumor periphery on CT (Fig. 3a). The nodules were increasing in size, ultrasound could not detect the nodule, and MRI could not be performed because of a pacemaker. Although preoperatively diagnosed as HCC by CT, the pathological findings of the surgically removed tumor indicated FNH (Fig. 3b). A non-cancerous liver specimen showed sinusoidal dilatation and mild fibrosis. Case 2: A 30-year-old male was detected with a hypervascular tumor on CT of the late arterial phase (Fig. 3c). The tumor was positive by PET-CT (Fig. 3e) and surgically removed, because TACE and PBT were ineffective. It was diagnosed as confluent-multinodular-type poorly differentiated-HCC based on liver cirrhosis (Fig. 3d). Two cases (2.6%) including case 1 were diagnosed as FNH by surgery. Therefore, the false-positive misdiagnosed rate was 2.6% (2 of 77 cases with nodules). It is difficult to take samples from the peripheral type of nodule. In one case, we performed a tumor biopsy just before transcatheter arterial chemoembolization (TACE) therapy to prevent bleeding. It is a method to diagnose HCC; however, it might increase the bleeding risk in patients in the margin. Therefore, new diagnostic method or marker for FALD-HCC is needed.

### Treatment and outcomes of HCC

The treatment of HCC is dependent on liver function and the number and size of tumors, establishing the various guidelines^60,61^. In addition to volumetry of HCC and residual liver, MELD-XI score and indocyanine green retention rate at 15 min (ICG-R15)^62^ may assist evaluation of liver function before surgery. In patients with FALD, cardiac function should be considered when selecting a treatment for HCC. There is limited evidence to suggest the optimal treatment strategy for FALD-HCC.

Liver resection increases the CVP if the IVC clumping is applied, and so was regarded as unsuitable for patients with FALD. Nemoto et al. performed surgery in the reverse Trendelenburg position without IVC clamping^63^. This procedure reduced the CVP from 12 to 10 mmHg without decreasing the systemic blood pressure resulted in reduced blood loss. Moreover, laparoscopic hepatectomy was reported as safe proceasear in the FALD setting^64,65^. The laparoscopic hepatectomy was safely performed keeping the pneumoperitoneum pressure at less than 6–10 mmHg and adequate fluid infusion was given to maintain cardiac preload^64^. Central venous pressure was monitored (11–21 mmHg) and end-tidal carbon dioxide tension was shifted to 36–40 mmHg^65^. The Pringle maneuver was applied during liver resection.

In contrast, the utility of radiofrequency ablation (RFA) is limited in the patients with a pacemaker, accumulation of ascites, and coagulopathy or anticoagulant therapy.

In previous studies, several cases were treated with transcatheter arterial chemo-embolization (TACE) or hepatic arterial infusion chemotherapy (HAIC)^6,56,66^ based on the greater hypervascularity of FALD-HCC (Table 4). The efficacy of TACE is limited in some cases of abnormal vasculature. We reported proton beam therapy (PBT) as a treatment option for patients with FALD-HCC^56,67^. PBT is potentially more beneficial in sparing organs-at risk^68^. For liver tumors, the tolerance of surrounding normal liver, biliary tracts, and gastrointestinal structures is the main limiting factor for dose escalation. Therefore, PBT has a dosimetric advantage compared to X-ray therapy. We treated four patients with HCC; no serious adverse event was observed^68^.

In addition to intrahepatic metastasis, most extrahepatic metastases are to the lungs. We experienced three cases of lung metastases among 12 FALD-HCC (Table 4). We speculated that the increase in pulmonary vascular resistance may reduce the blood flow speed, facilitating adhesion of cancer cells. This might promote the metastasis of HCC to the lungs.

### Risk factors for FALD and FALD-HCC

**Figure 4:**
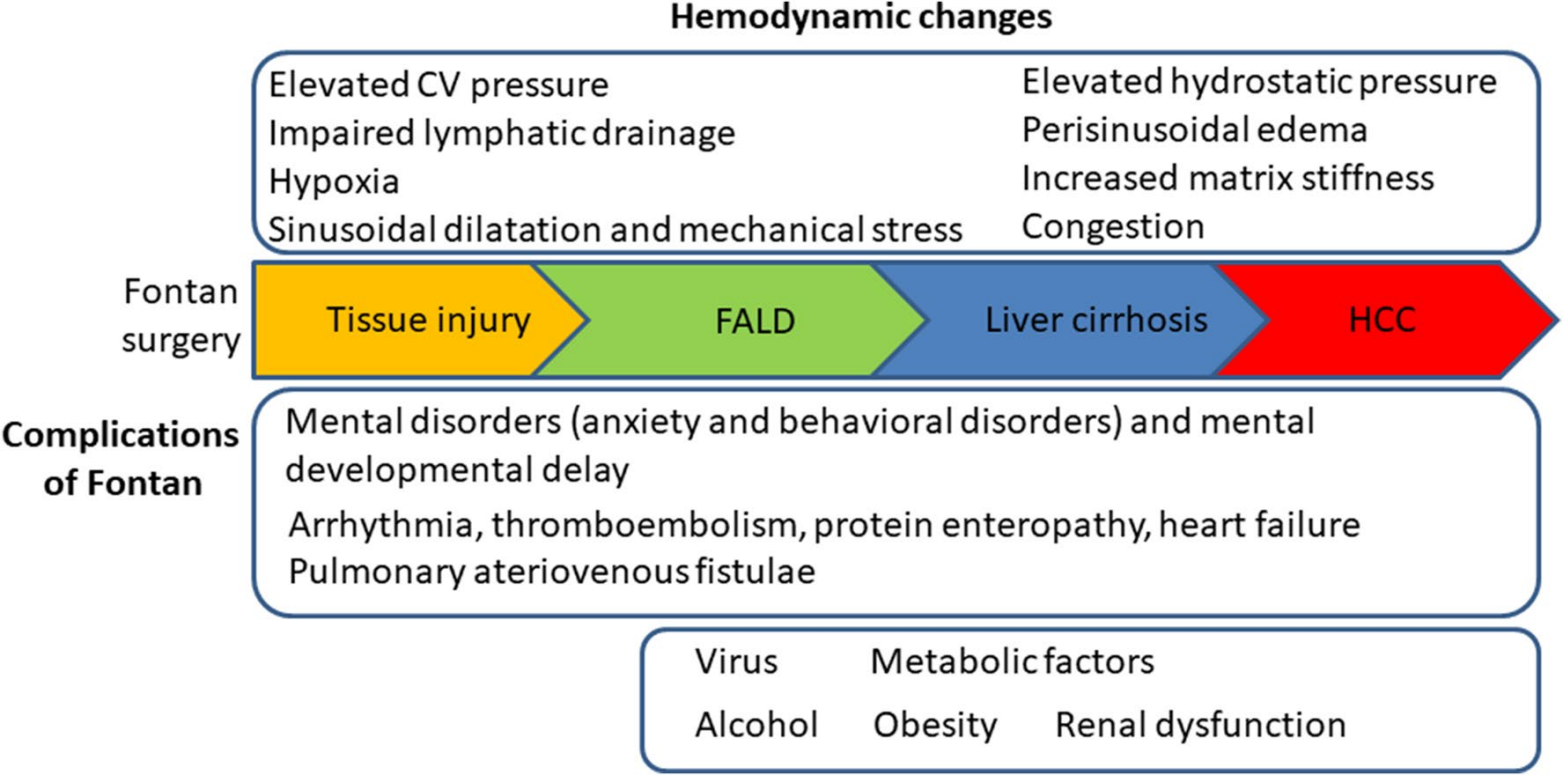
Development of fibrosis after Fontan surgery. Hemodynamic changes, complications of Fontan, viral infection, and metabolic factors are associated with the development of fibrosis. FALD, Fontan-associated liver disease; HCC, hepatocellular carcinoma.

The severity of fibrosis correlated with the duration of the Fontan procedure and the CVP^13,69^ (Fig. 4). In one case series, 43% of patients had advanced fibrosis 30 years after Fontan operation^51^. Additionally, aging, underlying hepatitis B or C infection, alcohol intake, and hepatotoxic drug use were associated with FALD development (Fig. 4). Timing of diagnosis, type of Fontan, cardiac complications, comorbid systemic disease and obesity may influence the clinical picture in ways that are poorly understood^14^.

Notably, cirrhosis is a strong risk factor for FALD-HCC^70^. The annual risk of HCC in cirrhotic patients with FALD was estimated to be 1.5–5.0%^6^. CVP has been reported to be 16.4 ± 6.1 mmHg in patients with liver cirrhosis after the Fontan procedure and 11.3 ± 2 mmHg in non-cirrhotic cases^13^. Although cirrhosis is a risk factor of HCC, it does not predict the prognosis. Ohuchi et al. reported that a high CVP and low arterial oxygen saturation strongly predict clinical events in children (*p* < 0.001), whereas these prognostic factors were marginal in adults^71^. Instead of CVP, renal dysfunction and metabolic abnormalities predicted clinical events in adults (*p* < 0.05). Therefore, medication and fenestration that lowers right atrial pressure are effective for decreasing CVP and might inhibit the progression of FALD; however, it may be insufficient to prevent FALD-HCC. The liver stiffness values on ARFI elastography were significantly higher in patients with hepatic nodules^31^. In our cohort, complications of polysplenia (HR 44.257, 95% CI 1.309–1495.862, *p* = 0.035) and higher FIB-4 index (HR 4.008, 95% CI 1.304–12.317, *p* = 0.015) were risk factors for FALD-HCC^56^.

### Surveillance of FALD and HCC

**Figure 5:**
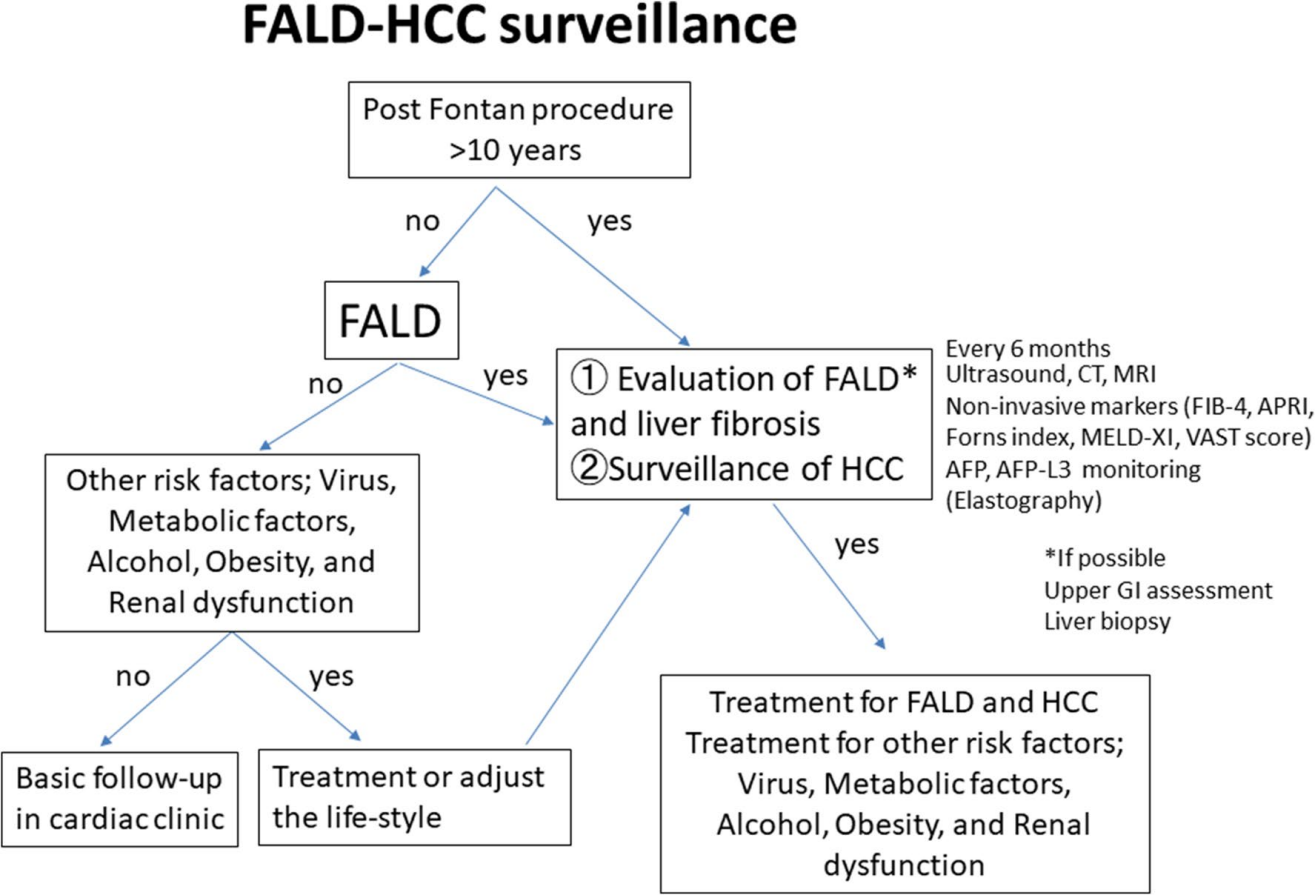
The algorithm for FALD-HCC surveillance. AFP, alpha fetoprotein, APRI, aspartate aminotransferase -to-platelet ratio index; CT, computed tomography; FIB-4, Fibrosis-4; FALD, Fontan-associated liver disease; GI, gastrointestinal; HCC, hepatocellular carcinoma; MELD, model for end-stage liver disease; MRI, magnetic resonance imaging, VAST score, varices, ascites, splenomegaly, thrombocytopenia.

A recent long-term follow-up study reported 10-, 20-, and 30-year survival rates of 74%, 61%, and 43%, respectively, among 1,052 patients after the Fontan procedure^72^. However, a recent systemic review of 65 FALD-HCC cases, which reported that 1-year survival is 50%^73^. Only four patients (6.2%) were under liver imaging surveillance for FALD-HCC, suggesting that HCC surveillance is necessary. There is no consensus on the surveillance of HCC in patients with FALD and the optimum screening method and interval are unclear^39^. In the presence of cirrhosis, serial monitoring by AFP and imaging every 6 months should be recommended similar to patients with HCV^74^. We think we could also follow the patients by this algorism to surveillance for FALD-HCC (Fig. 5). We experienced 12 cases of FALD-HCC, for an incidence of 0.8%, 2.9%, and 13.3% after 10, 20, and 30 years, respectively; these values are lower than those for HCV-related HCC^75^. The mortality rate of liver-related death among FALD is 0.19% (5/2,700 cases) and increased by ~ 29.4% (5/17 cases) in only those with liver cirrhosis and/or FALD-HCC (Overall, 25 year-survival rates after Fontan procedure were 68.6% and 97.9% in FALD-HCC and non-FALD-HCC, respectively, *p* < 0.01.)^49^. HCC must be diagnosed as at an early stage as possible to facilitate timely treatment. The incidence increases approximately 20 years after the operation. Therefore, we recommend that HCC surveillance should begin 10 years after the Fontan procedure^53,56,76^.

### Mental health care and transitional care of FALD in adults

People with a Fontan circulation have a higher rate of lifetime psychiatric diagnosis (65%) than their healthy peers (22%), particularly for anxiety and behavioral disorders^77^. Therefore, there needs to be a low threshold for the provision of mental health care. Also, from 21 to 76% of patients experience a break in regular follow-up cardiology care after transfer from pediatric to adult care^78^. Transitioning from child to adult care, including clinical and social care, is necessary for patients with FALD. FALD must be followed-up continuously for the lifetime of the patient.

## Conclusion and future perspective

The prevalence of FALD is increasing worldwide and the frequency of liver complications is rising because of improvement of cardiac survival. Evaluation of Fontan is different in children and adults, so further studies to identify non-invasive markers of fibrosis and FALD-HCC criteria are needed.

### Ethical approval

All procedures performed in studies involving human participants were in accordance with the ethical standards of the institutional and/or national research committee and with the 1964 Helsinki declaration and its later amendments or comparable ethical standards.

### Informed consent

Informed consent was obtained from the patient for the publication of our study.

## References

[1] Fontan F, Baudet E (1971). Surgical repair of tricuspid atresia. Thorax.

[2] d'Udekem Y (2007). The Fontan procedure: contemporary techniques have improved long-term outcomes. Circulation.

[3] Khairy P (2008). Long-term survival, modes of death, and predictors of mortality in patients with Fontan surgery. Circulation.

[4] Lindsay I, Johnson J, Everitt MD, Hoffman J, Yetman AT (2015). Impact of liver disease after the fontan operation. Am. J. Cardiol..

[5] Ghaferi AA, Hutchins GM (2005). Progression of liver pathology in patients undergoing the Fontan procedure: chronic passive congestion, cardiac cirrhosis, hepatic adenoma, and hepatocellular carcinoma. J. Thorac. Cardiovasc. Surg..

[6] Asrani SK, Warnes CA, Kamath PS (2013). Hepatocellular carcinoma after the Fontan procedure. N. Engl. J. Med..

[7] Shimizu H (2019). Thoracic and cardiovascular surgery in Japan in 2016: annual report by The Japanese Association for Thoracic Surgery. Gen. Thorac. Cardiovasc. Surg..

[8] de Leval MR, Deanfield JE (2010). Four decades of Fontan palliation. Nat. Rev. Cardiol..

[9] Weixler VHM (2020). Fontan with lateral tunnel is associated with improved survival compared with extracardiac conduit. J. Thorac. Cardiovasc. Surg..

[10] McRae ME (2013). Long-term issues after the Fontan procedure. AACN Adv. Crit. Care.

[11] Asrani SK (2012). Congenital heart disease and the liver. Hepatology.

[12] Deal BJ, Jacobs ML (2012). Management of the failing Fontan circulation. Heart.

[13] Kiesewetter CH (2007). Hepatic changes in the failing Fontan circulation. Heart.

[14] Daniels CJ (2017). Fontan-associated liver disease: proceedings from the American College of Cardiology Stakeholders Meeting, October 1 to 2, 2015 Washington DC. J. Am. Coll. Cardiol..

[15] Lautt WW (1985). Mechanism and role of intrinsic regulation of hepatic arterial blood flow: hepatic arterial buffer response. Am. J. Physiol..

[16] Ohuchi H (2016). Adult patients with Fontan circulation: What we know and how to manage adults with Fontan circulation?. J. Cardiol..

[17] Téllez L, Rodríguez-Santiago E, Albillos A (2018). Fontan-associated liver disease: a review. Ann Hepatol.

[18] Wu FM (2011). Liver disease in the patient with Fontan circulation. Congenit. Heart Dis..

[19] Baek JS (2010). Late hepatic complications after Fontan operation; non-invasive markers of hepatic fibrosis and risk factors. Heart.

[20] Wu FM (2017). Predictive value of biomarkers of hepatic fibrosis in adult Fontan patients. J. Heart Lung Transplant..

[21] Camposilvan S (2008). Liver and cardiac function in the long term after Fontan operation. Ann. Thorac. Surg..

[22] Fujisawa T, Tanaka Y (2013). Liver diseases in the fontan circulation. Pediatr. Cardiol Cardiac Surg..

[23] Shimizu M (2016). Risk factors and serological markers of liver cirrhosis after Fontan procedure. Heart Vessels.

[24] Kuno A (2013). A serum "sweet-doughnut" protein facilitates fibrosis evaluation and therapy assessment in patients with viral hepatitis. Sci. Rep..

[25] Kim TH (2018). Abdominal imaging findings in adult patients with Fontan circulation. Insights Imaging.

[26] Nakatsuka T (2019). Identification of liver fibrosis using the hepatic vein waveform in patients with Fontan circulation. Hepatol. Res..

[27] Wolff D (2016). The Fontan circulation and the liver: a magnetic resonance diffusion-weighted imaging study. Int. J. Cardiol..

[28] Nakajima K (2020). Visual liver assessment using Gd-EOB-DTPA-enhanced magnetic resonance imaging of patients in the early post-Fontan period. Sci. Rep..

[29] Wu FM (2014). Transient elastography may identify Fontan patients with unfavorable hemodynamics and advanced hepatic fibrosis. Congenit. Heart Dis..

[30] Sugimoto M (2016). Non-invasive assessment of liver fibrosis by magnetic resonance elastography in patients with congenital heart disease undergoing the Fontan procedure and intracardiac repair. J. Cardiol..

[31] Horvat N (2018). Multimodality screening of hepatic nodules in patients with congenital heart disease after Fontan procedure: role of ultrasound, ARFI elastography, CT, and MRI. AJR.

[32] Rathgeber SL (2020). Fontan-associated liver disease: spectrum of disease in children and adolescents. J. Am. Heart Assoc..

[33] Melero-Ferrer JL (2014). Fontan circulation in adult patients: acoustic radiation force impulse elastography as a useful tool for liver assessment. World J. Pediatr. Congenit. Heart Surg..

[34] Bradley CR (2018). Multi-organ assessment of compensated cirrhosis patients using quantitative magnetic resonance imaging. J. Hepatol..

[35] Johnson JA (2013). Identifying predictors of hepatic disease in patients after the Fontan operation: a postmortem analysis. J. Thorac. Cardiovasc. Surg..

[36] Kendall TJ (2008). Hepatic fibrosis and cirrhosis in the Fontan circulation: a detailed morphological study. J. Clin. Pathol..

[37] Schwartz MC (2013). Portal and sinusoidal fibrosis are common on liver biopsy after Fontan surgery. Pediatr. Cardiol..

[38] Komatsu H (2019). Liver disease secondary to congenital heart disease in children. Expert Rev. Gastroenterol. Hepatol..

[39] Hilscher M, Sanchez W (2016). Congestive hepatopathy. Clin. Liver Dis. (Hoboken).

[40] Malinchoc M (2000). A model to predict poor survival in patients undergoing transjugular intrahepatic portosystemic shunts. Hepatology.

[41] Heuman DM (2007). MELD-XI: a rational approach to "sickest first" liver transplantation in cirrhotic patients requiring anticoagulant therapy. Liver Transpl..

[42] Evans WN (2016). MELD-XI scores correlate with post-Fontan hepatic biopsy fibrosis scores. Pediatr. Cardiol..

[43] Berg CJ, Bauer BS, Hageman A, Aboulhosn JA, Reardon LC (2017). Mortality risk stratification in Fontan patients who underwent heart transplantation. Am. J. Cardiol..

[44] Gordon-Walker TT, Bove K, Veldtman G (2019). Fontan-associated liver disease: a review. J. Cardiol..

[45] Forns X (2002). Identification of chronic hepatitis C patients without hepatic fibrosis by a simple predictive model. Hepatology.

[46] McPherson S (2017). Age as a confounding factor for the accurate non-invasive diagnosis of advanced NAFLD fibrosis. Am. J. Gastroenterol..

[47] Elder RW (2013). Features of portal hypertension are associated with major adverse events in Fontan patients: the VAST study. Int. J. Cardiol..

[48] Zentner D (2020). Management of people with a Fontan circulation: a cardiac society of Australia and New Zealand Position statement. Heart Lung Circ..

[49] Kuwabara M (2018). Liver cirrhosis and/or hepatocellular carcinoma occurring late after the fontan procedure—a nationwide survey in Japan. Circ. J..

[50] Fukui H (2016). Evidence-based clinical practice guidelines for liver cirrhosis 2015. J. Gastroenterol..

[51] Pundi K (2016). Liver disease in patients after the Fontan operation. Am. J. Cardiol..

[52] Bryant R, Morales D (2018). Overview of adult congenital heart transplants. Ann. Cardiothorac. Surg..

[53] Téllez L (2020). Prevalence, features and predictive factors of liver nodules in Fontan surgery patients: the VALDIG Fonliver prospective cohort. J. Hepatol..

[54] Egbe AC (2018). Hepatocellular carcinoma after Fontan operation. Circulation.

[55] Johnson KB (2014). Advanced disease, diuretic use, and marital status predict hospital admissions in an ambulatory cirrhosis cohort. Dig. Dis. Sci..

[56] Sagawa T (2020). Characteristics of hepatocellular carcinoma arising from Fontan-associated liver disease. Hepatol. Res..

[57] Choi JY (2011). Focal nodular hyperplasia or focal nodular hyperplasia-like lesions of the liver: a special emphasis on diagnosis. J. Gastroenterol. Hepatol..

[58] Khan MA (2000). Positron emission tomography scanning in the evaluation of hepatocellular carcinoma. J. Hepatol..

[59] Wells ML (2017). Benign nodules in post-Fontan livers can show imaging features considered diagnostic for hepatocellular carcinoma. Abdom. Radiol. (NY).

[60] Kudo M (2014). JSH consensus-based clinical practice guidelines for the management of hepatocellular carcinoma: 2014 update by the liver cancer study group of Japan. Liver Cancer.

[61] Marrero JA (2018). Diagnosis, staging, and management of hepatocellular carcinoma: 2018 practice guidance by the American Association for the Study of Liver Diseases. Hepatology.

[62] Miyagawa S, Makuuchi M, Kawasaki S, Kakazu T (1995). Criteria for safe hepatic resection. Am. J. Surg..

[63] Nemoto S (2020). A patient with post-Fontan operation underwent left hepatectomy and caudate lobectomy for hepatocellular carcinoma: a case report. Surg. Case Rep..

[64] Angelico R (2019). Laparoscopic liver resection for hepatocellular carcinoma in Fontan-associated chronic liver disease. The first case report. Int. J. Surg. Case Rep..

[65] Yokota Y (2020). A case report of Fontan procedure-related hepatocellular carcinoma: pure laparoscopic approach by low and stable pneumoperitoneum. BMC Surg..

[66] Mazzarelli C (2019). Hepatocellular carcinoma as a complication of vascular disease of the liver after Fontan procedure. Hepatology.

[67] Ogasawara Y (2020). A case of Fontan-related hepatocellular carcinoma successfully treated with proton beam therapy. Clin. J. Gastroenterol..

[68] Mizumoto M (2011). Proton beam therapy for hepatocellular carcinoma: a comparison of three treatment protocols. Int J Radiat Oncol Biol Phys.

[69] Goldberg DJ (2017). Hepatic fibrosis is universal following fontan operation, and severity is associated with time from surgery: a liver biopsy and hemodynamic study. J. Am. Heart Assoc..

[70] Ioannou GN (2007). Incidence and predictors of hepatocellular carcinoma in patients with cirrhosis. Clin. Gastroenterol. Hepatol..

[71] Ohuchi H (2014). Comparison of prognostic variables in children and adults with Fontan circulation. Int. J. Cardiol..

[72] Pundi KN (2015). 40-year follow-up after the Fontan operation: long-term outcomes of 1,052 patients. J. Am. Coll. Cardiol..

[73] Rodríguez de Santiago E, Téllez L, Guerrero A, Albillos A (2020). Hepatocellular carcinoma after Fontan surgery: a systematic review. Hepatol. Res..

[74] Bruix J (2011). Maintenance therapy with peginterferon alfa-2b does not prevent hepatocellular carcinoma in cirrhotic patients with chronic hepatitis C. Gastroenterology.

[75] Sangiovanni A (2004). Increased survival of cirrhotic patients with a hepatocellular carcinoma detected during surveillance. Gastroenterology.

[76] Rychik J (2012). The precarious state of the liver after a Fontan operation: summary of a multidisciplinary symposium. Pediatr. Cardiol..

[77] DeMaso DR (2017). Psychiatric disorders in adolescents with single ventricle congenital heart disease. Pediatrics.

[78] Goossens E (2011). Transfer of adolescents with congenital heart disease from pediatric cardiology to adult health care: an analysis of transfer destinations. J. Am. Coll. Cardiol..

[79] Bae JM (2016). Fontan-associated liver disease: spectrum of US findings. Eur. J. Radiol..

[80] Josephus-Jitta D (2016). Three cases of hepatocellular carcinoma in Fontan patients: review of the literature and suggestions for hepatic screening. Int. J. Cardiol..

[81] Foucher J (2006). Diagnosis of cirrhosis by transient elastography (FibroScan): a prospective study. Gut.

[82] Kutty SS (2014). Increased hepatic stiffness as consequence of high hepatic afterload in the Fontan circulation: a vascular Doppler and elastography study. Hepatology.

[83] Diamond T, Ovchinsky N (2018). Fontan-associated liver disease: Monitoring progression of liver fibrosis. Clin. Liver Dis. (Hoboken).

[84] Ewe SH, Tan JL (2009). Hepatocelluar carcinoma—a rare complication post fontan operation. Congenit. Heart Dis..

[85] Saliba T (2010). Hepatocellular carcinoma in two patients with cardiac cirrhosis. Eur. J. Gastroenterol. Hepatol..

[86] Rosenbaum J, Vrazas J, Lane GK, Hardikar W (2012). Cardiac cirrhosis and hepatocellular carcinoma in a 13-year-old treated with doxorubicin microbead transarterial chemoembolization. J. Paediatr. Child Health.

[87] Elder RW, Parekh S, Book WM (2013). More on hepatocellular carcinoma after the Fontan procedure. N. Engl. J. Med..

[88] Wallihan DB, Podberesky DJ (2013). Hepatic pathology after Fontan palliation: spectrum of imaging findings. Pediatr. Radiol..

[89] Rajoriya N, Clift P, Thorne S, Hirschfield GM, Ferguson JW (2014). A liver mass post-Fontan operation. QJM.

[90] Weyker PD, Allen-John Webb C, Emond JC, Brentjens TE, Johnston TA (2014). Anesthetic implications of extended right hepatectomy in a patient with fontan physiology. A A Case Rep.

[91] Yamada K (2015). Transarterial embolization for pediatric hepatocellular carcinoma with cardiac cirrhosis. Pediatr. Int..

[92] Kwon S (2015). Surgical management of hepatocellular carcinoma after Fontan procedure. J. Gastrointest. Oncol..

[93] Oh C (2016). Hepatocellular carcinoma after the Fontan procedure in a 16-year-old girl: a case report. Medicine (Baltimore).

[94] Takuma Y (2016). Surgical resection for hepatocellular carcinoma with cardiac cirrhosis after the Fontan procedure. Intern. Med..

[95] Lo KS (2018). Left hepatectomy in a patient with a Fontan circulation. Transl. Gastroenterol. Hepatol..

